# Buffalo, Bush Meat, and the Zoonotic Threat of Brucellosis in Botswana

**DOI:** 10.1371/journal.pone.0032842

**Published:** 2012-03-08

**Authors:** Kathleen Anne Alexander, Jason Kenna Blackburn, Mark Eric Vandewalle, Risa Pesapane, Eddie Kekgonne Baipoledi, Phil H. Elzer

**Affiliations:** 1 Department of Fish and Wildlife Conservation, Virginia Tech, Blacksburg, Virginia, United States of America; 2 CARACAL (Center for Conservation of African Resources: Animals, Communities, and Land use), Kasane, Botswana; 3 Spatial Epidemiology & Ecology Research Laboratory, Department of Geography, University of Florida, Gainesville, Florida, United States of America; 4 Emerging Pathogens Institute, University of Florida, Gainesville, Florida, United States of America; 5 Botswana Department of Veterinary Services, Gaborone, Botswana; 6 Department of Veterinary Science, Louisiana State University Agricultural Center, Baton Rouge, Louisiana, United States of America; Global Viral Forecasting Initiative, United States of America

## Abstract

**Background:**

Brucellosis is a zoonotic disease of global importance infecting humans, domestic animals, and wildlife. Little is known about the epidemiology and persistence of brucellosis in wildlife in Southern Africa, particularly in Botswana.

**Methods:**

Archived wildlife samples from Botswana (1995–2000) were screened with the Rose Bengal Test (RBT) and fluorescence polarization assay (FPA) and included the African buffalo (247), bushbuck (1), eland (5), elephant (25), gemsbok (1), giraffe (9), hartebeest (12), impala (171), kudu (27), red lechwe (10), reedbuck (1), rhino (2), springbok (5), steenbok (2), warthog (24), waterbuck (1), wildebeest (33), honey badger (1), lion (43), and zebra (21). Human case data were extracted from government annual health reports (1974–2006).

**Findings:**

Only buffalo (6%, 95% CI 3.04%–8.96%) and giraffe (11%, 95% CI 0–38.43%) were confirmed seropositive on both tests. Seropositive buffalo were widely distributed across the buffalo range where cattle density was low. Human infections were reported in low numbers with most infections (46%) occurring in children (<14 years old) and no cases were reported among people working in the agricultural sector.

**Conclusions:**

Low seroprevalence of brucellosis in Botswana buffalo in a previous study in 1974 and again in this survey suggests an endemic status of the disease in this species. Buffalo, a preferred source of bush meat, is utilized both legally and illegally in Botswana. Household meat processing practices can provide widespread pathogen exposure risk to family members and the community, identifying an important source of zoonotic pathogen transmission potential. Although brucellosis may be controlled in livestock populations, public health officials need to be alert to the possibility of human infections arising from the use of bush meat. This study illustrates the need for a unified approach in infectious disease research that includes consideration of both domestic and wildlife sources of infection in determining public health risks from zoonotic disease invasions.

## Introduction

Brucellosis is a globally distributed disease caused by intracellular bacteria of the genus *Brucella*. Capable of infecting a wide variety of wildlife and domestic animal hosts, it is also one of the most widespread zoonotic diseases [1]. Animal infections most commonly occur through contact with infected fetal tissues and post-parturient discharges. Human infections occur from contact with infected animal tissues or ingestion of infected animal products [2]. Brucellosis has been documented in wildlife for nearly as long as the etiology has been understood [3], as for example, wood bison (*Bison bison athabascae*) in North America [4], [5], wild ungulates on the Iberian Peninsula [6] and eastern Spain [7], wild Saiga (*Saiga tatarica*) on the Kazakh steppe [8], and a number of wildlife species across Africa [9], [10], [11], [12], [13]. Despite a wide host range and broad distribution of this important pathogen, our understanding of its transmission and persistence dynamics are limited [14]. As with most multi-host pathogens, identification of reservoirs of infection can be complicated but this knowledge is critical to the successful development of management strategy directed at control or eradication of infection.

It is not known which species act as true reservoirs of infection for brucellosis. A reservoir is defined as one or more epidemiologically connected populations or environments in which the pathogen can be permanently maintained and from which infection is transmitted to a defined target population [15]. Presently, few true reservoirs of brucellosis, outside of cattle, have been identified [2]. While evidence for continued circulation of a pathogen in particular wildlife host species may be seen over time, pathogen persistence may not occur independently of livestock transmission pathways. For example, elk (*Cervus elaphus*) in the Greater Yellowstone Ecosystem (GYE) are able to maintain infection independent of livestock disease transmission with pathogen persistence in elk and bison populations threatening local livestock population health status [16]. In contrast, a recent study of red deer in Spain indicated that while the pathogen continues to circulate in the population, this species does not act as a reservoir of infection in that system [7]. Elimination of infection in domestic livestock resulted in pathogen fadeout in the red deer population. This illustrates the challenge and importance of careful evaluation of multi-host pathogen ecology where more in-depth study might be required to accurately identify transmission and persistence mechanisms critical to the development of effective control strategies.

McDermott and Arimi [17] reviewed brucellosis cases in livestock and humans across sub-Saharan Africa, including Botswana, and suggested that the disease was important but poorly understood in livestock and largely ignored in humans. As elsewhere in Africa, little is known regarding the dynamics and persistence of this disease in Botswana at the human-wildlife-domestic animal interface. This study describes a large-scale, retrospective assessment of *Brucella spp.* exposure among wildlife species and humans (reported cases) in Botswana in order to begin evaluating pathogen transmission and persistence dynamics in the country and their implications to both human and animal health.

## Results

### Serology

**Figure 1:**
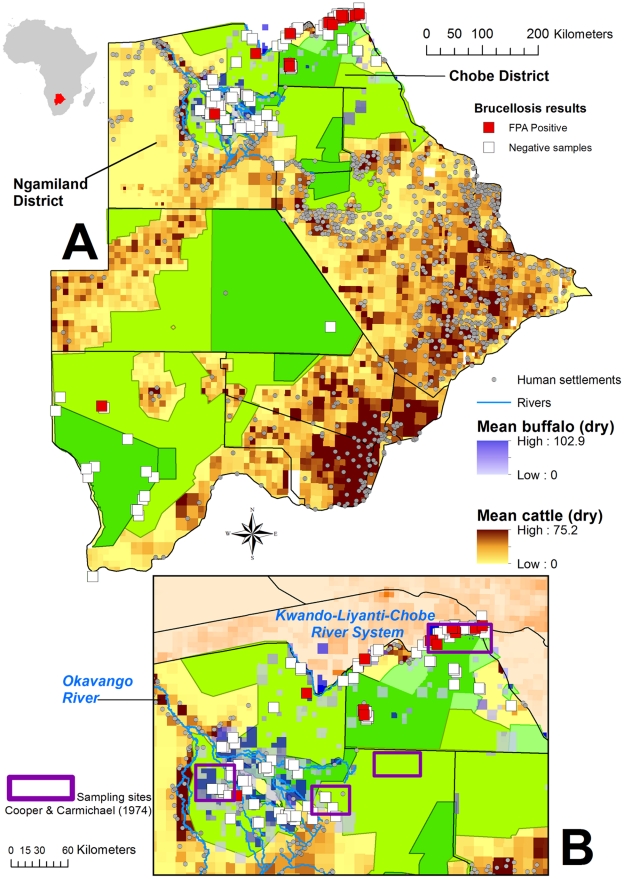
Mapped distribution of survey results for brucellosis antibodies among buffalo in Botswana in relation to average cattle counts (orange color ramp) and average buffalo counts (blue color ramp; Inset A); Inset B illustrates the distribution of FPA positive animals identified in this study (red squares) relative to historical samples screened by Cooper and Carmichael (1974; purple boxes). Green shaded areas are gazetted as conservation land use such as national parks, reserves, and wildlife management areas.

Of 46 samples positive on the Rose Bengal Test (RBT), 35% were confirmed positive by fluorescence polarization assay (FPA). African buffalo (6%, 95% confidence interval, 3.04%–8.96%, n = 247) and giraffe (11%, 95% confidence interval, 0–38.4%) were the only species where antibodies were serially detected on both the RBT and FPA test. Seroprevalence data from buffalo and giraffe are pooled here across years and sample areas, respectively, as there were no significant differences in seroprevalence levels after Bonferroni correction (Table 1). There was also no significant difference in seroprevalence levels between males and females among sampled buffalo where sex was known (χ^2^ p = .78, n = 206). Antibody positive buffalo were identified widely across the buffalo range in Chobe and Ngamiland Districts (Figure 1). Brucellosis antibodies were not detected in an isolated herd of buffalo found in Central District in 1999, outside the buffalo range. These buffalo were moved back to Ngamiland behind the buffalo fence for disease control purposes.

**Table 1:** Seroprevalence of brucellosis among sampled buffalo and giraffe by administrative districts and year of sampling (see Figure 3 for map of districts).

Species	District	Year	Present seroprevalence *(+/− 95% confidence limit)*	Total sampled
Buffalo	Chobe	1995	4% (0–14%)	27
		1996	13% (0–41%)	16
		1998	5% (0–15%)	43
		1999	7% (0–22%)	30
		2000	3% (0–10%)	39
	Ngamiland	1996	7% (0–19%)	86
		1998	17% (0–62%)	6
	Central	1999	0%	29
Giraffe	Chobe	1995	0%	1
	Ngamiland	1998	14% (0–39%)	7
	Southern	2000	0%	1

Human brucellosis was reported at low levels among patients presenting routinely at various government medical facilities across Botswana from 1974–1993 (37 cases, Table 2), 47% of infections reported during this period were in children less than 14 years of age.

**Table 2:** Human cases of brucellosis are presented by age, sex, occupation, and year of diagnosis.

Year	Cases	Occupation	Age Category				
			≤14 years	15–24	25–34	35–44	45–54
**1974**	15	ND	10 (F = 6, M = 4)	5 (F = 5)*			
**1975**	2	student	1 (F)	1 (F)			
**1977**	2	child & professor/technician	1 (M)	1 (M)			
**1982**	3	ND		1 (M)	1 (M)	1 (M)	
**1983**	2	ND	2 (M = 2)				
**1984**	2	ND			1 (M)		1 (M)
**1986**	1	ND				1 (M)	
**1987**	2	other	2 (F = 2)				
**1988**	2	other					2 (F = 2)
**1989**	2	other			2 (M = 2)		
**1990**	2	other	1 (M)		1 (F)		
**1993**	2	other				1 (M)	1 (M)
**Total**	**37**		**17**	**8**	**5**	**3**	**4**

Patients marked with (*) were identified as being ≥14 years of age and were, thus, grouped to the next highest age category. Categorical data choices included: professional/technician, administration, clerk, sale, service, agriculture, production, transport, labor, housewife, student, and child. ND denotes no available data.

### Buffalo Population Trends, Cattle densities, Seasonal Biomass, and Annual Change in Vegetation

**Figure 2:**
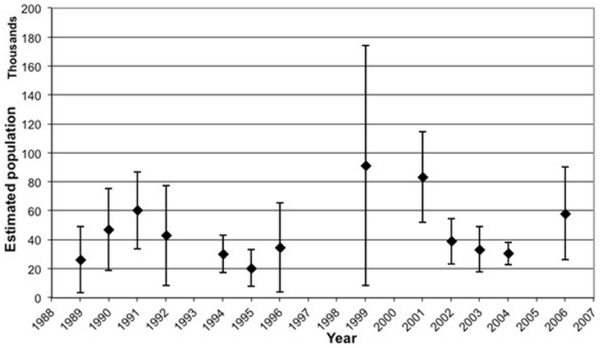
Estimated buffalo population numbers for Northern Botswana (1988–2006) from dry season aerial surveys conducted by the Department of Wildlife and National Parks.

**Figure 3:**
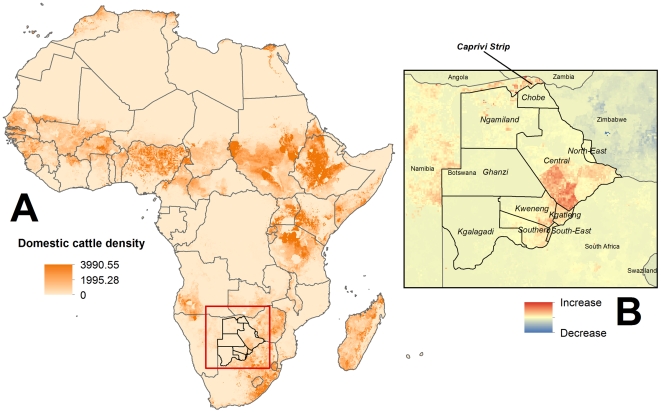
Cattle distribution mapped across Africa corrected to 2000 country estimates from the Food and Agricultural Organization (FAO) Gridded Livestock Production and Health Atlas (GLiPHA) livestock data set (Inset A) [50] **.** Changes in cattle density from 2000 to 2005 are illustrated in the Inset B (low to high, blue to red color ramp).

**Figure 4:**
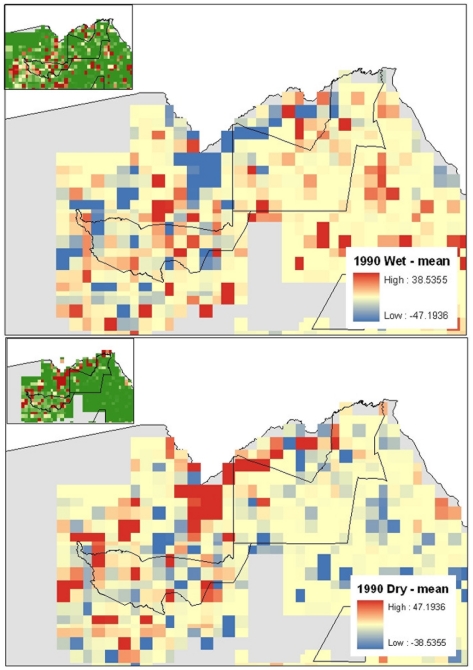
Seasonal changes in total biomass (livestock and wildlife combined) in northeastern Botswana from aerial animal surveys conducted by the Department of Wildlife and National Parks in the dry season (A-1990, B-1999) and wet season (C-1990, D-1999). Red cells represent increases in total biomass above the annual mean. Blue cells represent decreases below the annual mean. The green line represents the Chobe National Park boundary for reference. Note: the park is not fenced and wildlife populations occur throughout the area at different densities and intensity of overlap with humans.

**Figure 5:**
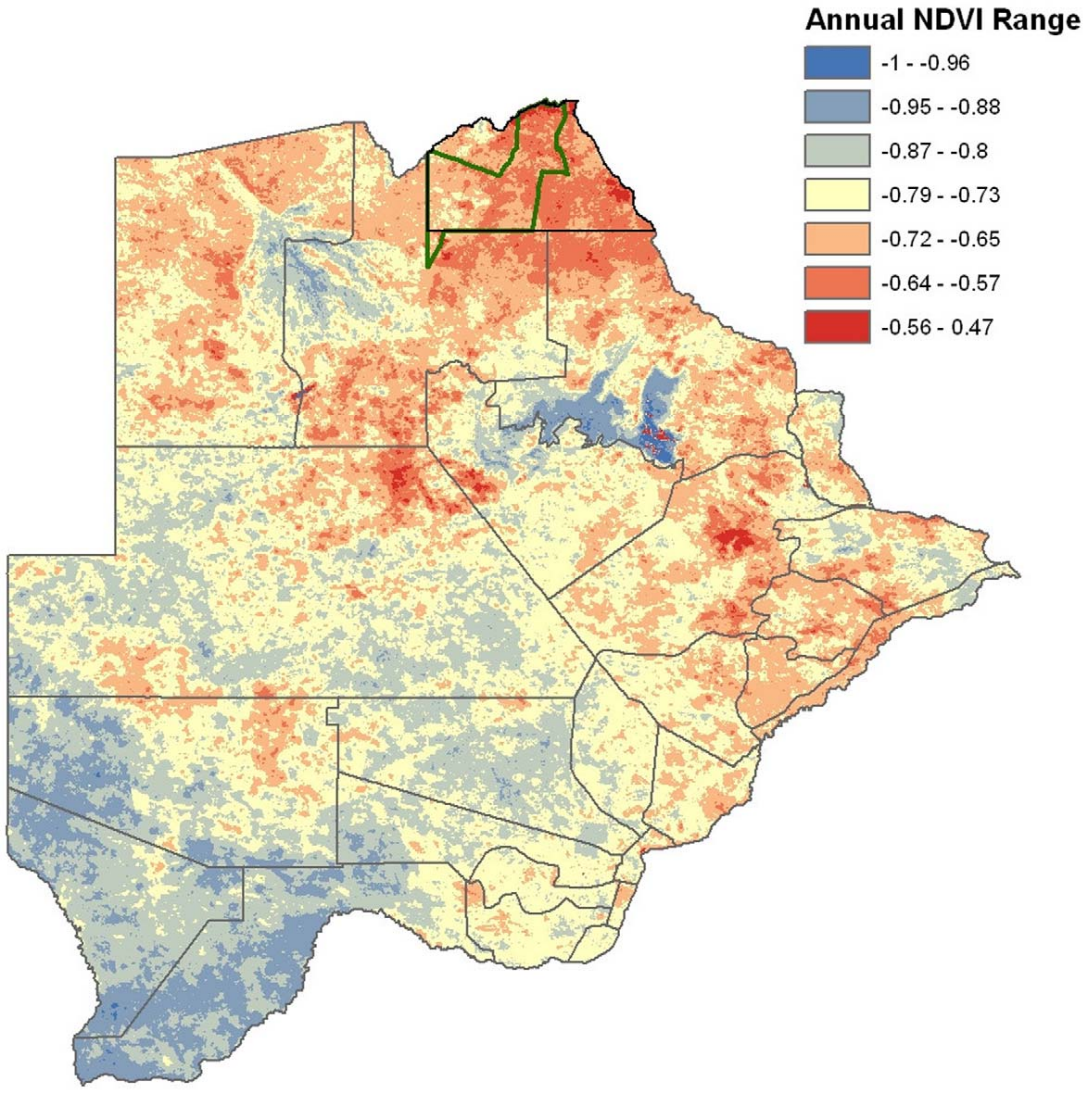
Map of Botswana showing annual range of greenness (NDVI) from temporal Fourier processed AVHRR satellite data illustrating the extreme variation in vegetation associated with rainfall, particularly in northern Botswana. Red areas indicate zones with the most dramatic change in vegetation over the year. The green area indicates the location of Chobe National Park (referred to in Figure 4).

The estimated buffalo population of northern Botswana appears to have fluctuated over the last 2 decades and, despite high confidence limits for each survey, numbers increase and decrease with a mode of 4 to 5 years from lows of about 20,000 to highs of between 60,000–80,000 (Figure 2). Cattle estimates from the Food and Agricultural Organization (FAO) for the whole of Africa are presented in Figure 3 and local BASIS-derived estimates for Botswana are presented in Figure 1. Strong seasonal effects on the geographic distribution of biomass density (livestock and wildlife combined) were identified (Figure 4) with the highest density of animals during the dry seasons concentrated along the riverfronts. In contrast, during the wet season, animals are spread out across the landscape. These seasonal shifts are most pronounced in and around Chobe District, where the effects of seasonal water availability is most dramatic on vegetation (Figure 5).

## Discussion

This work in Botswana updates the global survey of Pappas *et. al.* [1] identifying the persistent presence of brucellosis in buffalo and the occurrence of human brucellosis infections in the country from 1970–1993. We provide an overview of brucellosis diagnostics and approaches utilized in this field study and then discuss brucellosis ecology in buffalo and local human communities, previous research in the area, and the potential role of bush meat in human pathogen exposure and brucellosis infection.

While isolation and culture of *Brucella* bacteria are identified as the gold standard for determination of brucellosis, surveillance and control programs are generally undertaken using indirect serologic testing approaches [18]. RBT is often used as an initial screening test, as it is highly sensitive and able to pick up chronic infections in ruminants [19]. However, the specificity of RBT is relatively low [19], requiring a confirmatory test with greater specificity. FPA has been identified as a highly sensitive and specific (99% and 99%, respectively) diagnostic tool in previous field trials in cattle [20].

Using RBT for screening and FPA for confirmation, we identified the presence of brucellosis-specific antibodies in buffalo and giraffe in northern Botswana from wildlife collected over a relatively long time period (1995–2000) and across a large spatial area (Figure 1). In this study, the FPA was easily adapted to a remote field setting and performed well under such conditions, as has been reported elsewhere [20], [21].

Despite screening a large number of African ungulates and predators, only buffalo (6%) and giraffe (11%) were positive for antibodies to brucellosis on both the RBT and FPA serologic tests (Figure 1). Others have found *Brucella* antibody positive giraffe despite a similarly limited number of giraffe evaluated [22]. The clinical significance of this finding is unknown as is the potential for diagnostic cross-reactions with other brucellosis strains, including the possibility that there is an unknown giraffe-specific strain.

In Botswana in the 1970's, brucellosis was considered to be widespread in cattle and goats with 17% of cattle antibody positive among sampled clinically normal animals [13]. With active veterinary control measures in place, such as vaccination, outbreaks of clinical disease in livestock appear to be reduced with disease reports involving a low number of cattle cases annually (n = 1–18 individuals, 1996–2004, OIE reports, Handistatus 2, http://www.oie.int/hs2/). African buffalo, sampled in the 1970s in similar areas as this study (see Figure 1B) [13] identified 11% of sampled buffalo (n = 233) antibody positive on serum agglutination tests (this study 14% RBT positive). *Brucella* specific antibodies were not detected in any other wildlife species sampled. While different serologic tests were used in the 1970s study and in the present evaluation, there appears to be a relatively low but consistent level of brucellosis seroprevalence among buffalo over this time period, suggesting an endemic status [23].

In Botswana, buffalo populations are separated in the north from the high density cattle production areas in the southeastern part of the country through a system of cordon fences in order to control the potential threat of livestock diseases such as foot and mouth disease. Botswana buffalo populations can, however, freely mix with buffalo and cattle within the Caprivi area in Namibia across the Kwando-Chobe-Linyanti River system (Figures 1,3). Brucellosis outbreaks have been reported in livestock in Namibia at low levels similar to that noted for Botswana [17]. Madsen and Anderson [10] have suggested that the buffalo is a reservoir host for brucellosis in Zimbabwe. Given low levels of overlap with cattle through most of the buffalo range in northern Botswana, with the exception of the Caprivi, and widespread evidence of infection across their range on both surveys (Figure 1), buffalo in Botswana may also act as a reservoir of infection independent of livestock transmission, but this cannot be conclusively determined at present.

In this system, the multiplicity of potential hosts complicates the determination of the role of any individual host species or population in pathogen transmission dynamics and persistence. To determine if buffalo act as a true reservoir of infection independent of livestock sources, it would be necessary to eliminate all transmission between buffalo and other host species in the system and demonstrate that buffalo are capable of sustained infection independent of transmission from other infection reservoirs [15]. In the absence of being able to achieve this largely implausible scenario, new molecular epidemiological tools and strain differentiation offer promise and can contribute importantly to understanding pathogen transmission and persistence among species and hosts across the landscape [24].

Infectious disease dynamics are often strongly influenced by seasonal patterns, irrespective of pathogen transmission mode [25]. The breadth and consistency of these patterns suggest that seasonal influences on host and pathogen biology can have significant effects on patterns of pathogen invasion and transmission [26]. The relationship between host abundance and pathogen transmission, influenced strongly by seasons in semi-arid environments, is central to understanding infectious disease ecology and patterns and processes of pathogen invasion. Botswana provides an important example of this potential influence with extreme seasonal climatic variation, which occurs within and between years. There are only two perennial sources of water in the northern buffalo range: the Okavango and the Kwando-Linyanti-Chobe Rivers with surface water outside of these systems being ephemeral. Extreme wet and dry seasons within the year (see Figure 5), and extended multi-year cycles of dry and wet periods occur over time and strongly influence density and distribution of animals and potential contact between species in the system. In the dry season in Chobe District, for example, animal densities are concentrated along the Kwando-Linyanti-Chobe Rivers while during the wet (rainy) season animals disperse across a vast region following the newly available growth in vegetation and water in rain filled pans (Figure 4). As water resources are exhausted in the interior at the end of the wet season, water dependent wildlife populations return to the only permanent water resource in the system, the Kwando-Chobe-Linyanti River. Animal numbers concentrate dramatically along the river during this time, a resource that is shared with the local human populations in the region. Similar movements of domestic animals are observed but at a much finer scale.

Brucellosis transmission operates as a function of host density [16]. In the GYE, high rates of infection in elk were associated with winter-feeding of herds and increased herd density at artificial feeding grounds [27]. Seasonal fluctuations in water availability in northern Botswana may replace or mimic the feeding ground dynamic of the GYE by concentrating animals and increasing density (Figure 4). Water availability is highly variable in time and space in relation to rainfall patterns and this can strongly influence density and spatial distribution of domestic animals and wildlife including buffalo over the whole year including buffalo calving periods. Under climate change, Botswana is predicted to become drier by 5 to 15% per century [28]. Increasing restriction of water resources will further concentrate water dependent species and brucellosis transmission potential might be expected to increase in the region.

Bovine brucellosis can cause abortion, stillborn calves, retained placentas, and, infrequently, male infertility [29]. What is the conservation importance of brucellosis infection in buffalo? Buffalo populations in Botswana appear to have fluctuated over the last 2 decades; the reason for this pattern is unknown but may be due to wet and dry cycles across years as well as changing policy regarding utilization (1988–2006, Figure 2). Previous studies in Uganda and Tanzania have found similar buffalo population fluctuations [30] with low levels of brucellosis. Infectious diseases that primarily impact reproduction are thought to be more likely to exert population regulatory effects than those pathogens that primarily cause mortality [23]. The effects of brucellosis on population dynamics of infected wildlife species are unclear, although previous studies have attempted to unravel these interactions [4]. However, it is important to recognize that the population effects of infectious disease are context dependent, an outcome of complex interactions between the host, pathogen(s), environment, and other sources of mortality that may be influencing host demographics (poaching, hunting, etc.), which are expected to be location specific. The population effect of brucellosis in Botswana's buffalo is unclear at present.

With the exception of a higher year of reported cases in 1974, brucellosis was diagnosed at very low levels among human patients presenting at various medical facilities across the nation (1974–1993, 37 cases, Table 2). There were no agricultural workers identified among human-case reports. Most of the recorded infections were identified in children less than 14 years of age. Previous studies have found brucellosis in children uncommon but consumption of raw milk was identified as the primary source of infection in these cases [31]. In Botswana, children will also assist in processing of meat from animal slaughter for household or ceremonial use with the role in these activities largely determined by the gender of the child. Children can be given certain parts of the carcass based on birth order, for example, or other culturally driven traditions [32]. It is not clear if the reported number of human cases of brucellosis extracted from these national government reports accurately reflects the occurrence of human infection in the country, as there have been no systematic studies of the disease in the population. Under reporting is possible as infection is often misdiagnosed due to a lack of knowledge about the pathogen by health professionals [33]. This may be related to the expectation by health professionals that the risk of human infection is minimal based on known levels of livestock disease further complicated by difficulty in definitively diagnosing infection and the lack of appropriate laboratory support should the physician suspect infection. Diagnosis may be further complicated and masked by the myriad of other infectious diseases that can have a similar clinical presentation [34].

The impact of Human Immunodeficiency Virus/Acquired Immune Deficiency Syndrome (HIV/AIDS) on brucellosis transmission is unknown. A limited assessment of brucellosis infections in HIV/AIDS patients did not find a causal association despite the fact that HIV/AIDS victims are more sensitive to intracellular pathogens [35]. However, with 68% of the world's HIV cases [36], and widespread occurrence of brucellosis across the continent, there is a need to develop a better understanding of the impact of HIV on the epidemiology and transmission dynamics of brucellosis at the human-animal interface in Africa.

Given that brucellosis appears to be well controlled in livestock in a particular region, it might be expected then that the potential for human infection would be of reduced importance in public health surveillance strategy. However, bush meat is consumed and handled (legally and illegally) by local populations living in the buffalo and giraffe range and so human exposure from these wildlife sources is possible.

The history of buffalo and giraffe utilization in Botswana is long and part of an extensive culture of wildlife product use for food, raw material, and social and ceremonial purposes [37], [38]. Buffalo are considered prized bush meat in Botswana and are utilized preferentially as the opportunity presents. For example, buffalo meat is considered to produce the most superior biltong (dried meat) over any other source of meat including that derived from livestock sources [39]. Preference for buffalo bush meat is similar in most African countries where buffalo occur [40], [41]. This preference for buffalo meat is further observed in the smuggling of buffalo and other wildlife meat into developed nations such as France to supply African immigrants who prefer such meat to locally available products [39].

Historically in Botswana, the chiefs of the various tribes controlled hunting of buffalo, giraffe, and other wildlife species even under British colonial rule. With independence in 1966, and the establishment of the Fauna Conservation Act, the Botswana Government established Special Game Licenses (1979) allowing rural communities to continue to utilize wildlife and legally hunt buffalo and other wildlife in remote areas. This was done to ensure that people dependent on bush meat had continued legal access to this resource [37], [42]. Special Game Licenses for buffalo and other wildlife species were also issued to the Botswana Defense Force and to community groups in fire-fighting exercises in remote areas within the buffalo range. Buffalo were even given to communities for cooking and feasting at Independence Day celebrations but later this practice was stopped (Alexander pers. obs.). Today, buffalo are still utilized under permits provided by the Department of Wildlife and National Parks (DWNP) through a quota system based on population numbers. Meat from buffalo killed by trophy hunters can be given to communities resident in the respective concession area where it might be freely distributed or sold by community-based organizations involved in managing the hunting concession [43]. Botswana citizens can also legally hunt buffalo under a Single Game License distributed through a raffle system based on adequate buffalo population numbers. Buffalo are also an important conflict species and can be legally killed by communities or DWNP officers when the animal is considered a threat to human life or property (Republic of Botswana Conservation and National Parks Act, 2001). Destroyed buffalo are slaughtered on site with the help of community members and sold under auction *in situ* to discourage the killing of wildlife and community expectation of access to free meat resulting from wildlife destruction. Illegal taking of buffalo also occurs throughout the buffalo range (DWNP, unpublished data) as with other wildlife. Giraffe are also poached in Botswana for both meat and medicinal purposes (DWNP unpublished data) [44].

Processing of raw meat and animal products can expose humans to brucellosis infection through cuts and abrasions in the skin [45]. While men normally undertake the slaughtering of animals, the whole family can be involved in post-slaughter handling of the butchered carcass exposing members, including children, to blood and raw animal products [32]. Varying local traditions and culture will control roles in the process and distribution of meat to members of the family and community including elderly relations [39].

While the zoonotic disease risk of brucellosis might be considered limited in countries where the disease is well controlled and regulated in livestock, bush meat utilization, practiced in Botswana and over much of Africa, identifies an alternate human exposure risk. It is important, however, to note that the threat of bush meat as a zoonotic source of disease transmission is not restricted to Africa alone. Increasing use of bush meat in the developed world and globally has increased our recognition of bush meat associated zoonotic threats, as for example, with the use of feral swine meat and brucellosis infection in hunters in the United States and Australia [46]. There is, therefore, an important need to identify bush meat consumption patterns, preferences, and pathogen presence in order to determine the full spectrum of zoonotic pathogen transmission risk associated with the use of these products.

While it is known that wildlife can be important in brucellosis transmission dynamics, lack of data has meant that wildlife may not be explicitly included in models used to evaluate animal and public health control strategies [47]. Wildlife present a complex component of transmission that can be difficult to characterize and there is a need for surveillance data to be coupled with molecular, genetic, and dynamical modeling tools in order to begin to unravel this complexity.

### Conclusions

This study indicates that buffalo may be an important species contributing to pathogen transmission dynamics and persistence in southern Africa, acting not only as a potential source of infection to livestock but also as a direct zoonotic pathogen threat to humans in areas where buffalo occur and are consumed. Our results indicate that human health facilities in Botswana and elsewhere in Africa should be alert to the potential for brucellosis infection where bush meat is consumed and wildlife sources of infection may occur. Directed research is needed to identify the regional profile of zoonotic disease threats that may potentially arise where human consumption of bush meat is practiced.

It is recommended that potential wildlife hosts should be systematically included in brucellosis surveillance even in the presence of minimal or non-existent livestock case reporting. Molecular genetic tools and dynamical modeling should be integrated with strategic disease surveillance to identify transmission and persistence dynamics in these potential multi-host pathogen systems.

Brucellosis remains a globally important zoonotic disease affecting both human and animal populations. This paper highlights the importance of taking a unified approach in infectious disease research that includes consideration of both domestic and wildlife sources of infection in determining public health risks from zoonotic disease invasions. Increasing our understanding of the ecology of this zoonotic pathogen will be critical to both human and animal health particularly in regions where reservoirs of infection may not be well characterized or are simply unknown.

## Materials and Methods

### Ethics Statement

No human subjects work was undertaken in this study; human brucellosis case data were extracted from annual government reports. These government reports are prepared public reports, providing summarized count data of patients diagnosed at Government hospitals by category of disease and year. All data were anonymised.

All animal samples used in this study were obtained from archived collections of Dr. K.A Alexander. This study did not involve any capture of live animals but only access to archived materials. Archived samples accessed for this study originally collected from live animals were done so humanely in consideration of the welfare of the animals in full accordance of the laws of Botswana and through the approval and supervision of the Directorate of the Department of Wildlife and National Parks under the Botswana Government. To avoid any stress and to engage humane treatment, all live wild animals sampled for various departmental activities were chemically immobilized under the supervision of a Botswana-registered veterinarian, according to species-specific protocols, in most cases, by the Department of Wildlife and National Parks, Wildlife Health Unit Head, who at that time was Dr. K.A. Alexander. As a Botswana Government employee at the time, Dr. K.A, Alexander would not have been given a permit or approval identification number.

### Study Site

Botswana is a landlocked country in sub-Saharan Africa where vast parts of the country are dry with access to only ephemeral sources and borehole reticulated water. There are only three perennial sources of water throughout the country: the Limpopo, Okavango, and Kwando-Linyanti-Chobe Rivers (international boundary between Botswana and Namibia) and a handful of dams at primary city centers. Botswana has three distinct seasons that strongly influence the movement of wild and domestic animals: the wet season (December–April), the cool, dry season (May–August), and the hot, dry season (September–November). In order to control livestock disease transmission, a series of veterinary cordon fences have been erected. Buffalo populations in the country are consequently restricted to the northern part of the country separated from the primary cattle export and buffer zones. Over 37% of the country's land area is gazetted as protected for wildlife, supporting large and diverse wildlife populations, which vary in composition by habitat type and water availability. Within the structure of veterinary cordon fences, wildlife movement is unrestricted across protected and unprotected land use types in both the northern and southern parts of the country.

### Serum sample collection

Serum samples used in this study were collected from various sub-adult and adult wildlife species sampled across Botswana in a variety of land uses (e.g., protected areas, state, and tribal land) in conjunction with ecological and health research and as necropsy assessment of culled or naturally dying animals (Figure 1, 1995–2000). Wildlife species tested included African buffalo (*Syncerus caffer*, n = 247), bushbuck (*Tragelaphus scriptus*, n = 1), eland (*Taurotragus oryx*, n = 5), elephant (*Loxodonta africana*, n = 25), gemsbok (*Oryx gazelle*, n = 1), giraffe (*Giraffa camelopardalis*, n = 9), red hartebeest (*Alcelaphus buselaphus*, n = 12), impala (Aepyceros melampus, n = 171), greater kudu (Tragelaphus strepsiceros, n = 27), red lechwe (*Kobus leche*, n = 10), reedbuck (*Redunca arundinum*, n = 1), white rhino (*Ceratotherium simum*, n = 2), springbok (*Antidorcas marsupialis*, n = 5), steenbok (*Raphicerus campestris*, n = 2), warthog (*Phacochoerus aethiopicus*, n = 24), waterbuck (*Kobus ellipsiprymnus ssp. defassa*, n = 1), common wildebeest (*Connochaetes taurinus*, n = 33), honey badger (*Mellivora capensis*, n = 1), lion (*Panthera leo*, n = 43), and Burchell's zebra (*Equus quagga ssp. burchellii*, n = 21).

Human disease data were extracted from annual health reports prepared by the Central Statistics Office from data compiled by the Ministry of Health (1974–2006) under the Botswana Government. Reports reflect the summary diagnoses of patients attending government hospitals and clinics across the nation over the respective year.

### Brucellosis serologic testing

Antibodies to *Brucella spp.* were detected using the RBT, (Cenogenics, Morganville N.J.) according to previously published procedures [18]. Samples testing positive on RBT were then confirmed with FPA as previously described [48] using a commercial *Brucella* antibody test kit (Meridian Life Sciences Incorporated, Memphis TN). FPA positive animals were mapped in relation to all sampled animals and long-term average buffalo and domestic cattle data.

### Buffalo Population Trends, Domestic Cattle Distribution, Seasonal Biomass, and Annual Change in Vegetation

Buffalo population trends were derived from aerial surveys conducted by the Department of Wildlife and National Parks and mapped using ArcGIS v10 (ESRI, Redlands, CA). Estimates were generated from the BASIS program (Botswana Aerial Survey Information System, Version II, Government of Botswana) based on the Marriott 4-cell Method [49]. Gridded GIS layers of average buffalo and cattle populations were created in the Spatial Analyst Extension of ArcGIS 10.

To evaluate the relationship between cattle in Botswana and neighboring countries, we used the gridded livestock data of the world for 2000 and 2005 [50]. The 2000 data were used to represent cattle distributions for the time period of wildlife sampling. To evaluate trends in cattle population density for the region around Botswana, we subtracted the 2000 raster from the 2005 raster using Spatial Analyst.

We mapped seasonal change using temporal Fourier processed Normalized Difference Vegetation Index (NDVI) data from the Advanced Very High Resolution Radiometer (AVHRR) satellite sensor provided by Hay et al. [51]. We evaluated the range of inter-annual NDVI by subtracting the annual minimum NDVI from the maximum NDVI using the raster calculator in the Spatial Analyst extension. To illustrate the seasonal fluctuation of available water and change in wildlife and livestock densities and distribution across the country, we mapped the seasonal change in total biomass (livestock and wildlife combined) from the BASIS dataset by subtracting the annual mean from the wet and dry season estimates of total animals in each grid cell for two different years.
